# Study the behaviour of Ti-Mo-xZr alloys during thermomechanical treatment

**DOI:** 10.1038/s41598-026-45667-y

**Published:** 2026-04-13

**Authors:** Alaa Keshtta, Hayam A. Aly, Gamal Abd ELnaser, Tharwat Elsarag, Shaban Abdou, Ahmed H. Awad

**Affiliations:** 1 Department of Production Engineering and Mechanical Design, Faculty of Engineering, Port Said University, Port Said, 42526 Egypt; 2 Central Metallurgical Research and Development Institute (CMRDI), Helwan, 11421 Egypt; 3 Design and Production Department, Faculty of Engineering, Ain Shams University, Cairo, 11535 Egypt

**Keywords:** Ti-Mo-Zr alloys, Bo-Md diagram, Thermo-Calc, Bio-implants, Thermo-mechanical treatment, Engineering, Materials science

## Abstract

Titanium and its alloys became the master materials for bio-implants, possessing perfect stability inside the human body and excellent mechanical properties over traditional implant materials. Continuous development of commercial implant alloys, including Ti-6Al-4 V, results in approaching new Ti-alloys that are more biocompatible with the human body, replacing Al and V elements with vital alternatives. In this study, the influence of Zr addition to Ti–10Mo–xZr (x = 0, 3, 6 wt%) alloys during the thermomechanical processing on the phase stability, mechanical properties, and electrochemical behavior is investigated. Thermo-Calc software, which showed that Zr addition to Ti-10Mo reduces β-transus temperature and represents Zr as a β-stabilizer element. DSC experimental results agreed with the software calculations, approving Zr addition to minimize the α to β transformation temperature.XRD and SEM results revealed β phase as predominant in Ti–10Mo (94.6%) and Ti–10Mo–6Zr (82.3%), while Ti–10Mo–3Zr alloy exhibited (52.5%) α-phase, with non-linear phase evolution with Zr content due to the dual effect of element addition and thermomechanical processing. The elastic modulus ranged from 108.9 to 120.4 GPa, with Ti–10Mo–3Zr achieving the lowest modulus. The scoped alloys achieved high compressive ductility (> 60%), which gives a good mechanical computability. Electrochemical evaluation in simulated body fluid revealed composition-dependent corrosion behavior, where β-rich alloys demonstrated enhanced polarization resistance, while microgalvanic effects in the Ti-10Mo-3Zr alloy increased corrosion susceptibility. The corrosion rates in simulated body fluid are $0.21843 \times \, 10^{ - 3}$, $4.5714 \times \, 10^{ - 3}$, and $0.642 \times \, 10^{ - 3}$ mm/year for Ti-10Mo, Ti-10Mo-3Zr, and Ti-10Mo-6Zr alloys, respectively.

## Introduction

Over the last two decades, Titanium alloys have widely invaded the biomedical product market because of their attractive properties as a remarkable lightweight metal that possesses a low strength-to-weight ratio, besides a low Young’s modulus. At high temperatures up to 530 °C, titanium alloys achieved high corrosion resistance as well as compatibility with biological systems. Comparing both cobalt-based alloys and stainless-steel corrosion resistance values with titanium and its alloys, titanium has higher qualities and stability, adapting to bio-implant applications^1–3^. Titanium alloys bio-implant applications examples are such as cardiology, orthopedics, dentistry, and neurosurgery^4^.

Titanium alloys mainly consist of at least one of two equilibrium α or β phases, which are preferred to stabilize depending on the added element. These elements are divided into α-stabilizing elements, β-stabilizing elements, and neutral elements^5–7^. In addition, non-equilibrium phases can be achieved from previous equilibrium phases through either α' hexagonal or α″orthorhombic martensite structures. The formation of entire non-equilibrium phases can be achieved by controlling alloy cooling rates, plus the amount of β phase. The higher presence of β phase results in the appearance of α″orthorhombic martensite rather than α' hexagonal in the quenched conditions^8^. Besides the alloying element, to enhance the material characteristics of bio-implants, electromagnetic treatments were approved to have a significant influence on the alloys^9^. The manufacturing process also plays a role in the Ti-alloys properties, as previous work studied the Ti powder, focusing on α-phase precipitations^10^.

Experimental and clinical investigations have demonstrated that a mismatch in elastic modulus between implants and bones leads to a “stress shielding” phenomenon. The low elastic modulus is subjected to less stress when two materials with different elastic moduli are stressed simultaneously^11^. Wolff’s law^12^ suggests that if the bones of the human body are exposed to long-term external pressure, the density and hardness of the bone may increase. However, bones will become osteoporotic and resorptive if they are exposed to low stress or remain stress-free for an extended period. For the minimization or prevention of such an impact, scientists are committed to the production of alloys with a low elastic modulus. Ti and its alloys are the most studied and the most promising to be used as hard tissue implant materials^11^.

The growing studies on Ti-6Al-4 V have focused on the manufacturing processes utilizing the additive metals process to enhance the performance^13,14^. Even though Ti-6Al-4 V, a type of α + β Ti-alloy, has been found to have a negative impact due to its lower biocompatible alloying elements. These elements release ions inside the human body into the bloodstream, potentially leading to health issues. Also, debris separated from the implant is released into surrounding tissues, further raising concerns. Such contaminants, whether ions or debris, have been associated with an increased risk of Alzheimer’s and metabolic bone diseases^15,16^. Jayasheelan V. and etal^17^ When they studied the surface chemistry of Ti-6Al-4 V alloy, they recognized aluminum oxide, which would lead to ion metal dissolution, as used for long-term implants.

The growing need for v-free Ti-alloys approaching better biocompatibility rather than Ti-6Al-4 V. β-type Ti alloys have increased the focus on their potential to reduce the stress shielding effect between human bone and implant due to their low modulus of elasticity^18,19^. Human bones average elastic modulus range (7–30 GPa) and α + β Ti alloys elastic modulus range (~ 110 GPa)^19^. Researchers went on to study the behavior of additional β-stabilizing elements to Ti-alloys, seeking accepted mechanical properties and biological adaptation^20^. Intensive studies were conducted to investigate Ti-Nb^21,22^, Ti-Mo^23–25^, Ti-Ta^26^, Ti-Mn^27^, Ti-Nb–Ta^28,29^, Ti-Nb-Zr^30^, Ti-Mo-Zr^31,32^, Ti-Mn-Zr^33^, Ti-Ta-Zr^34^, Ti-Nb–Ta-Sn^35^, Ti-Nb-Zr-Sn^36^ systems, and more, willing to compete with competitive bio-implant alternatives. Another advantage is that Mn, Mo, and Fe elements are low-cost elements that can be used to produce cost-effective alloys^37^.

Molybdenum (Mo) is a key alloying ingredient used to create Ti alloys with low elastic modulus for implant biomaterials. Ti alloys, Mo is a common β stabilizer. For a variety of applications, numerous researchers have designed and developed new Ti alloys with low modulus Ti alloys using the Mo equivalent^38^. Molybdenum acts as a β-stabilizing element in titanium alloys and has become a research interest because it has lower toxicity than vanadium. Numerous studies have approached favorable mechanical compatibility as well as cytocompatibility of titanium alloys with molybdenum addition such as Ti–Mo^39,40^, Ti–Mo–Zr^41,42^, Ti–Mo–Ta^43^, Ti–Mo–Si^44^, Ti–Mo–Fe^45^, Ti–Mo–Zr-Sn^46^ and Ti–Mo–Zr–Fe^23^ alloys. Lin et al.^31^ Obtained a higher yield strength of Ti-Mo-Zr by Zr element addition, and the elastic modulus increased due to the strengthening mechanism achieved by dissolved Zr.

The zirconium element shows allotropic transition like titanium, HCP to BCC transus temperature transformation occurs at 862 °C^37^. Zr achieved growing attention as a role candidate for biomedical applications because of (a) the Surface formation of apatite layer, bone-like inside the human body, (b) excellent biocompatibility as well as bio-corrosion resistance, and (c) better mechanical properties^47^. In the periodic table, the atoms Zr and Ti are in the same group. Consequently, they share a lot of physical and molecular traits. Numerous researchers have investigated the phase shift, microstructure, and characteristics of titanium and its alloys concerning a particular quantity of Zr addition, leading to the development of a wide range of TiZr-based alloys with exceptional strength and endurance^11^.

The amount of ions released by Ti-Zr alloys is much less than in the case of pure Ti, which proves the higher stability of the ZrO_2_ layer compared to TiO_2_^37^. Also, another advantage of Zr addition to Ti-alloys was reported by Pinghua et al., in which they achieved greater cytocompatibility of Ti-Zr alloy, exceeding CP-Ti^48^. Yuhua Li et al.^49^ studied the corrosion resistance upon the addition of the Zr element to Ti-alloys with the influence of heat treatment. They found that Zr addition obtained a higher passive film, resulting in significant corrosion resistance. Also, Zr addition had improved the Ti-alloys’ wear resistance due to the formation of the hard ZrO2 oxide film, which is a promising result for biomedical alloys^50^.

The current study aims to explore new vanadium-free alloys incorporating non-toxic elements, non-allergenic, and relatively good cost-effective properties, explicitly focusing on biomedical applications. Mo selection as an alloying element with Ti in this work was to utilize biocompatible materials requirements, attain desirable low Young’s modulus plus mechanical qualities, and achieve good cost-effectiveness. This work is an extension of previous studies that discussed the effect of the Zr addition to β-type Ti-14Mn-xZr alloy with percentages 0Zr, 3Zr, and 6Zr, which concluded that Zr lowered the elastic modulus, giving suitable properties for bio-implant applications^51^. Based on the output of this study, the same Zr gradient was applied to a common Ti-10Mo bio-implant alloy. The theoretical calculation of alloy design of the current study was published, including the allocation of $\overline{Bo }-\overline{Md }$ and $\stackrel{-}{e/a}$—Δ $\overline{r }$^52^. The theoretical analysis deepens in the current paper as phase stability is discussed later using Thermo-Calc software.

## Experimental work

**Fig. 1:**
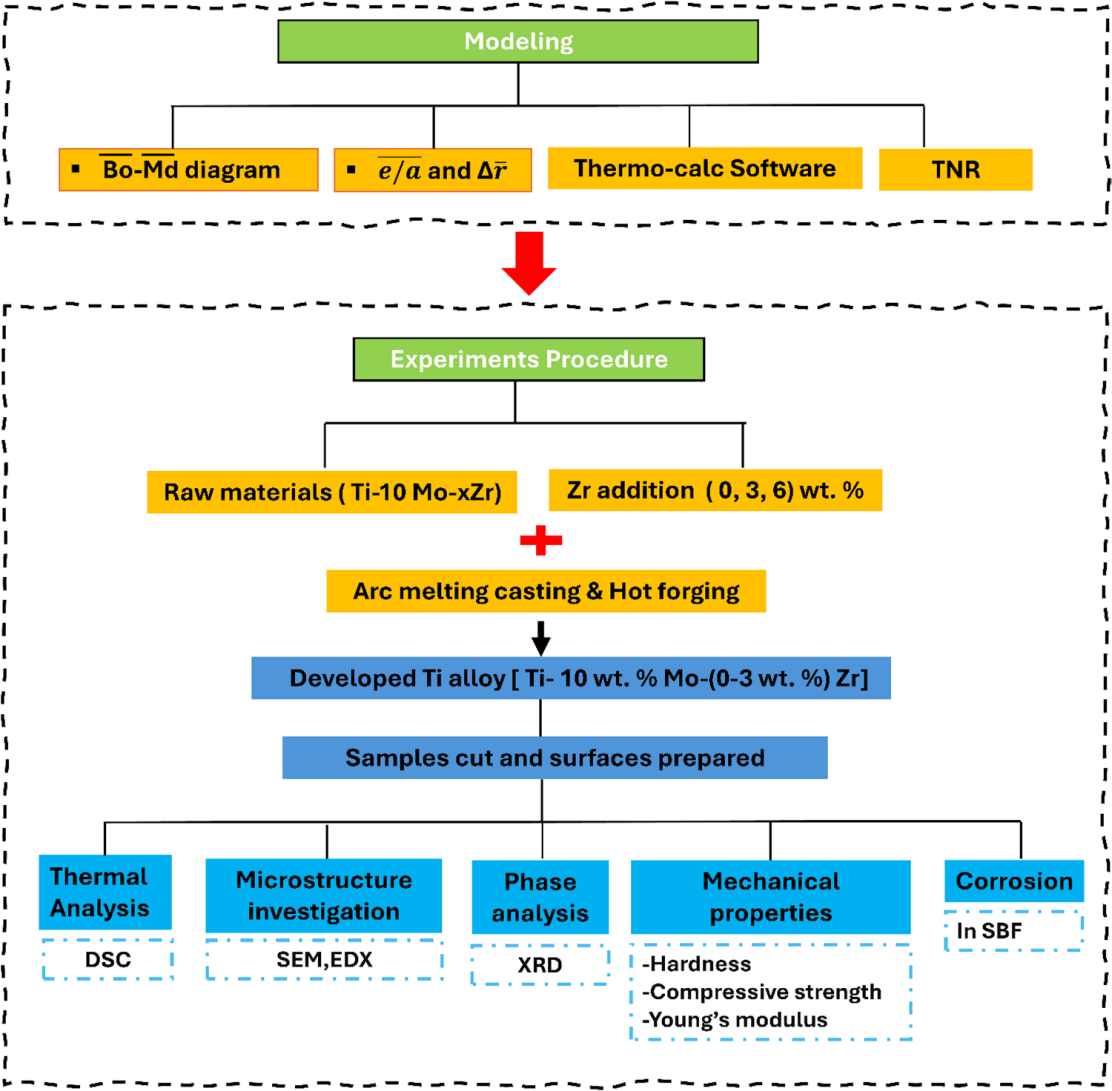
Schematic drawing for modeling and experimental framework.

The experimental procedures employed in the present study were according to the materials, equipment, and tests. To achieve the objectives of this study, a description of the initial materials, along with the methodologies, tools, and configurations employed for the assessment of microstructural, mechanical properties, and corrosion in simulated body fluid, is provided. The magnetic properties and corrosion behavior in saline solutions of entire alloys were investigated in previous studies^52,53^, and this study is an extension of those characteristics. The framework of the study, modeling, and experimental work is presented in Fig. 1.

### Alloy design

Trial and error have been the mainstay of the development process since the inception of the systematic study of Ti-alloys and their use in biomedical applications. Overcoming the obstacle of having a comparatively higher elastic modulus than human bone was the main issue. It makes intuitive sense that the key to customizing the desired mechanical properties of titanium and its alloys is to manage the internal structure. Over the last ten years, new low-cost, low Young’s modulus, and biocompatible Ti-alloys have been designed by utilizing first-principles calculations and the d-electron theory of alloy design. Titanium alloys with a lower E-modulus are systematically designed using the d-electron alloy design theory by utilizing two electronic parameters, namely $\overline{Bo }$ (covalent bond strength between Ti and alloying elements) and $\overline{Md }$ (d-orbital energy). The assessment of the overall stability of the α and β phases is conducted by studying alloying elements using Mo equivalent concentrations ([Mo]eq). The alloying element contribution is expressed in the following equation. The alloys allocation in $\overline{Bo }-\overline{Md }$ and $\stackrel{-}{e/a}$—Δ $\overline{r }$ were published in a previous study^52,54^.

$$\begin{aligned} \left( {Moeq} \right)_{Q} & = \, 1.0 \, Mo \, + \, 1.25 \, V \, + \, 0.59 \, W \, + \, 0.28 \, Nb \, + \, 0.22 \, Ta \, + \, 1.93 \, Fe \, + \, 1.84 \, Cr \, + \, 1.51 \, Cu \\ & + \, 2.46 \, Ni \, + \, 2.67 \, Co \, + \, 2.26 \, Mn \, + \, 0.30 \, Sn \, + \, 0.47 \, Zr \, + \, 3.01 \, Si \, - \, 1.47 \, Al \, \left( {wt \, pct} \right) \\ \end{aligned}$$

However, it has been established that a minimum molybdenum (Mo) equivalent content of around 10 wt.% is necessary in order to achieve the stabilization of the beta phase during the rapid cooling process of titanium-based alloys^55^. Previous studies focused on Ti-10Mo alloy as a biocompatible solution for bio-implants and investigated the different heat treatment effects on the phase stability^56–58^. In this study, Ti-10Mo-xZr alloys were investigated, henceafter abbreviated by TMZx, where x is the composition of Zr element. Table 1 includes the alloy compositions used and the theoretical calculations of different parameters.

**Table 1:** Alloy compositions used in the current study, with their code and different parameter calculations.

Alloy code	Alloy composition (wt.%)			$\overline{Bo }$	$\overline{Md }$	$\stackrel{-}{e/a}$	Δ $\overline{r }$	Mo_eq_
	Ti	Mo	Zr					
TMZ0	Bal	10	0	2.804	2.421	4.105	−0.147	0.1
TMZ3	Bal	10	3	2.81	2.429	4.107	0.069	0.1138
TMZ6	Bal	10	6	2.815	2.437	4.108	0.293	0.1276

### Material preparation

High-purity sponge titanium, molybdenum, and zirconium were used to produce the current studied alloys; these elements were weighed by digital balance with accuracy up to the third digit of a gram. The metals were cleaned using distilled water, followed by ethanol, in an ultrasonic device. All the alloys were produced using an electric arc melting furnace in an argon atmosphere, maintained at a pressure of 700 mbar. To ensure the removal of any contaminants, the chamber underwent triple evacuation cycles, reaching a pressure of 5 × 10^–2^ mbar, followed by flushing with argon. The maximum utilized current was 400 A, and the ingot was kept in the liquid state for 20 s and subsequently solidified into a button-shaped ingot. Each sample was flipped to be re-melted twice inside the furnace and finally produced ingots, each of which is ~ 100 g.

Each ingot was homogenized in a muffle furnace at 900° C for 1.8 Ks, then hot forged, followed by water quenching. During the solidification process of Ti-based alloys from elevated temperatures above β-transus, the cooling rate is the main factor controlling phase transitions^59,60^. Both cooling rate and alloy composition influence the solid phases that emerge during the phase transformation process. These phases may encompass β → α as achieved in the current study. The ingots were cut into smaller specimens using a wire cutting machine.

### Material testing

#### Phase identification

Phases were detected using X-ray crystallography (Shimadzu-6100) equipped with a Cu-Kα target with a secondary monochromatic beam at 45 kV and 40 mA. The samples examined at 2Ө range from 30° to 90°, and the step size was 0.04°. The card number (PDF 01–086–2610) was used to detect the β phase, and (PDF 01–077–3481, PDF 01–077–3484) was used for detecting the α phase.

#### Microstructural analysis

The microstructure was investigated using higher magnification images obtained using scanning electron microscopy (TESCAN MIRA), equipped with an electron backscatter detector, and energy dispersive X-ray spectroscopy (EDX) for elemental analysis, where the results were plotted using the attached type (OXFORD XPLORE).

#### DSC analysis

Differential scanning calorimetry (DSC) analysis was conducted using a NETZSCH STA 409 thermal analyzer. Samples were heated in an argon atmosphere up to 650 °C with a heating rate of 10 °C/min.

#### Hardness

The hardness was studied using (INNOVATEST, NEMESIS 9140, 200 g-3000 kg) Vickers hardness tester with a load of 3 kg_f_ for 15 s at room temperature. Five reads for every condition were reported, then the highest and the lowest values were extracted, and an average of three median reads was calculated.

#### Compression

The compression tests were conducted using a 100-ton universal testing machine (UH-F1000KNI, SHIMADZU) at room temperature. Samples were cut into a cylinder shape, whose length is parallel to the forging direction, with dimensions (⌀5 mm and 8 mm length) using a wire cutting machine. The compression load was applied at a constant rate of 0.1 mm/min and parallel to the forging direction.

#### Elastic modulus

The elastic modulus was measured using an Olympus EPOCH 600- digital ultrasonic flaw detector. The Young’s modulus measurement depends on the ultrasonic method (UT), in which the wave velocities are obtained by the pulse-echo technique.

A longitudinal transducer working at 4 MHz is used to generate longitudinal velocity. Specimens should be contacted with the longitudinal transducer in the presence of Olympus Couplant Glycerin. Conversely, a shear transducer working at a frequency of 2 MHz used for shear velocity measurement, specimens should contact the shear transducer in the presence of Couplant Olympus/shear Gel 54-T04. Applying Archimedes’ method, the specimens’ density was measured, and wave transit time was obtained during longitudinal and shear transducer usage. The wave transits through the specimens’ thickness for the operated samples, with dimensions of (10 mm × 10 mm × 8 mm). All specimens were ground up to grit 4000 and polished until a mirror surface for both parallel surfaces of the measured specimens, and the average of ten measurements was taken. The following equations are used for longitudinal and shear ultrasonic wave calculations.

$$V_{L} = 2l/t_{L}$$

$$V_{S} = 2l/t_{S}$$

where: *V*, *V*_*S*_ are the longitudinal and shear ultrasonic wave velocities, respectively. The *t*, *t*_*S*_ parameters are the longitudinal and shear ultrasonic wave transit time, respectively. The following explains the equations used for elastic modulus calculations in this study:

$$E \, = \, 2G \, (1 + \nu )$$

where: $$G \, = \, Shear \, modulus \, = \rho V_{S}^{2}$$, $$\rho = \, density \, of \, alloys$$, $$K = \, Bulk \, modulus \, = \, L - \frac{4}{3}G$$, and $$v = Poisson^{\prime}s \, ratio \, = \, \left( {L - 2G} \right)/2(L - G)$$.

#### Corrosion tests

The electrochemical parameters of the current studied alloys through simulated body fluid were measured using an AUTOLab PGSTAT 30 potentiostat, with a three-electrode setup. The counter electrode was Platinum foil, and the reference electrode was a saturated calomel. The immersion time of the studied alloys was 15 min in the simulated body fluid, while in the open-circuit potential examination, the stabilization was observed after 10 min. The simulated body fluid composition is presented in Table 2.

**Table 2:** Simulated body fluid content.

Chemical	Content (g/L)
NaCl	8
NaHCO_3_	0.35
NaH_2_PO_4_.2H_2_O	0.06
MgSO_4_.7H_2_O	0.06
CaCl_2_	0.14
KH2PO4	0.06
KCl	0.4
glucose	1
MgCl2 ·6H2O	0.1

## Results and discussion

### Equilibrium phase stability

**Fig. 2:**
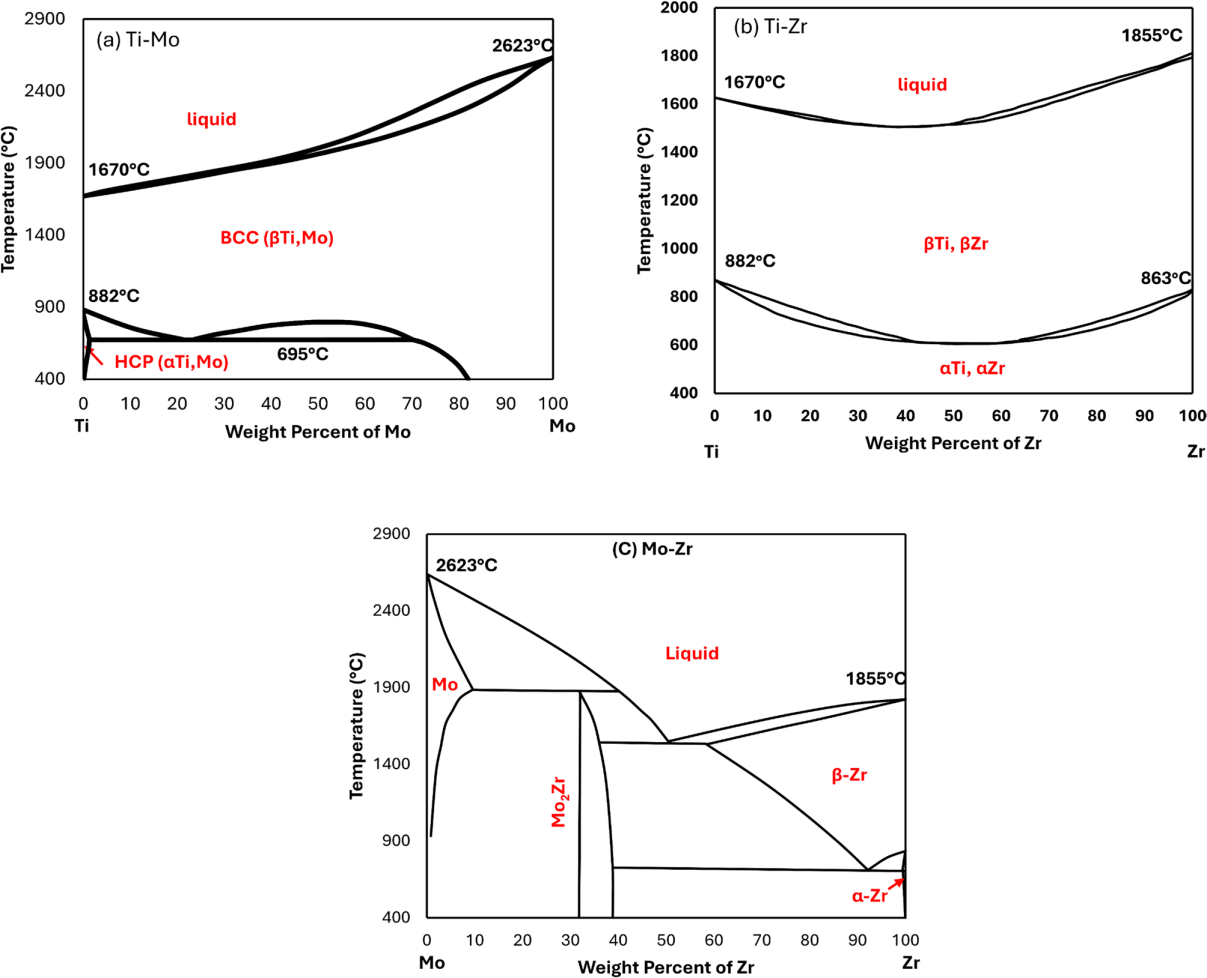
Binary phase stability diagrams of (**a**) Ti-Mo, (**b**) Ti-Zr, and (**c**) Mo-Zr^62^. Permission was taken from Elsevier.

Thermo-Calc software produced phase diagrams of ternary alloys; the ternary equilibrium of Ti-Mo-Zr alloys depends on binary phase diagrams for Ti-Mo, Ti-Zr, and Mo-Zr thermodynamic assessments. Hence, binary phase diagrams are crucial for understanding the behavior of the material, as permitted by Elsevier, the binary phase diagrams are redrawn in Fig. 2. From literature studies, molybdenum is proven to be a low-cost β-stabilizing element in Ti alloys^61^. The binary phase stability diagram of Ti-Mo is plotted in Fig. 2a has a eutectoid reaction, including the α-Ti (HCP) and β-Ti (BCC) phases, and no intermetallic compound or brittle phases appeared. Melting points for pure elements of Ti and Mo are 1670 °C and 2623 °C, respectively; the Mo addition to Ti caused increases in the melting temperature of the entire alloy. The composition of the current study, Ti-10Mo alloy, is located in the hypo eutectoid region and exhibits upon cooling α-Ti (HCP) and β-Ti (BCC) phases.

The binary phase stability diagram of Ti-Zr alloys is shown in Fig. 2b. The Zr pure element has a melting point of 1855 °C. The phase diagram includes three different phases: liquid, high-temperature α (HCP) phase, and β (BCC) stable at room temperature; the Ti-Zr phase diagram has no intermetallic compound reaction. Titanium, a pure element, has a melting point of 1670 °C. Zr additions to Ti have variant effects on the melting temperature of the alloys. Adding Zr to Ti decreased the melting point to approximately 1550 °C at around 50 wt%, and superior Zr addition increased the melting points. β/α transition temperatures have a similar trend as melting points, in which the minimum transition point occurs at 627 °C and 65 wt.% of Zr.

The equilibrium phase stability of the Mo-Zr alloying system is shown in Fig. 2c, revealing three invariant reactions: intermetallic compound, eutectic, and eutectoid. The intermetallic compound Mo_2_Zr appeared at 32 wt.% of Zr and up to a temperature of 1880 °C. A eutectic reaction occurs at 50.5 wt% Zr and 1545 °C, the liquid phase transfers into β-Zr (BCC) and C15_LAVES phase (Mo_2_Zr). The last reaction, eutectoid decomposition of β-Zr (BCC) solid solution into the α-Zr (HCP) phase and C15_LAVES phase (Mo_2_Zr), occurs at 92 wt% Zr and 713 °C. At high Zr content, more than 58%, the Mo-Zr phase diagram emerged with complete solubility of the β-Zr phase. Although at low Zr content below 0.65 wt. % showed a limited solubility of the Mo phase into α-Zr (HCP).

### Thermodynamic calculation (Thermo-Calc)

**Fig. 3:**
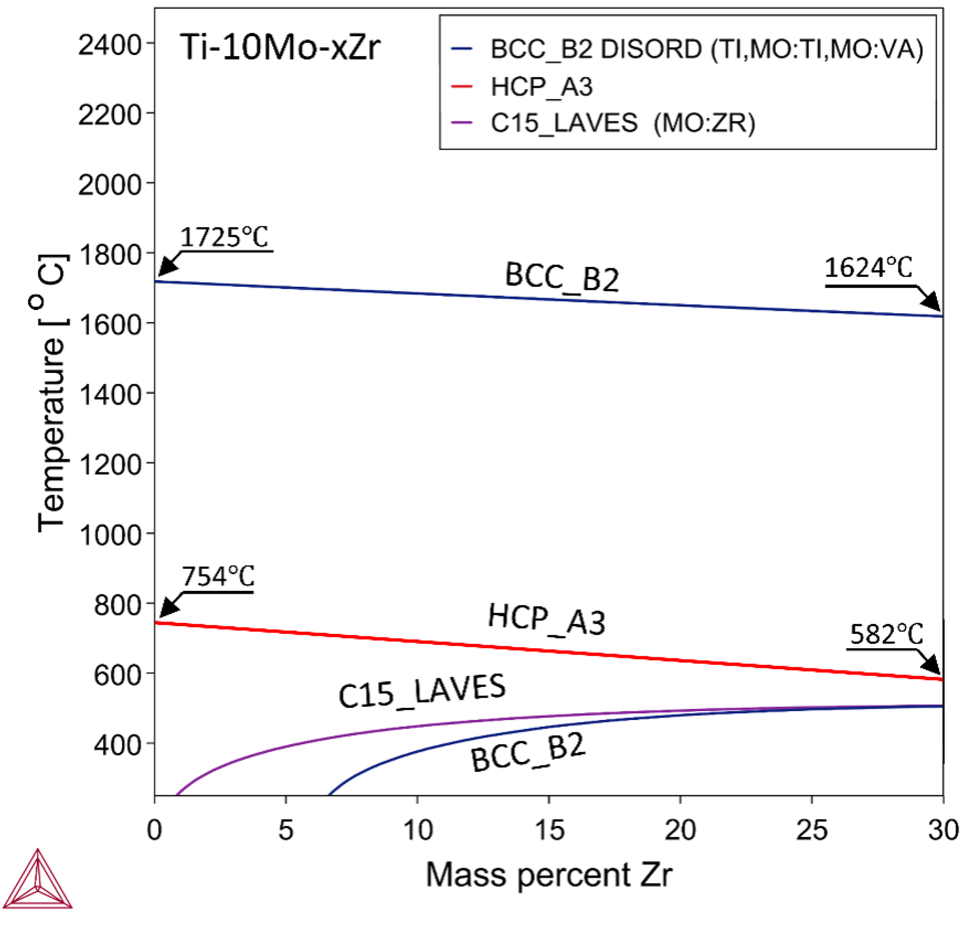
Phase diagram calculations using Thermo-Calc software and TCTI2 database for Ti–10Mo–(x)Zr alloys.

Thermo-Calc software was used to predict transformation kinetics through thermodynamic calculations of the currently investigated alloys of Ti-10Mo-xZr. In Fig. 3, the effect of Zr content on phase stability in a range of (x = 0–30 wt%) alloyed with Ti-10Mo-xZr at different temperatures up to 2400 °C using the TCTI2 database. This phase diagram showed three invariant phases: β (BCC), α (HCP), and C15_LAVES (Mo_2_Zr intermetallic compound). BCC_B2 solidus line slope slightly decreased inversely with the Zr element addition through Ti-10Mo-xZr alloys. Increasing the Zr mass percentage results in a lower β-transus temperature observed by B2 line inclination, which confirms the effect of Zr addition as a β-stabilizing element. Phases transition during liquid → BCC transformation occurred at 1725 °C and 1624 °C for 0Zr and 30Zr wt%, respectively, with a reduction ≈ of 100 °C. These results agree with previous claims that Zr acts as a weak β stabilizer element in Ti alloys^63^. HCP_A3 line incline decreased as the Zr element was added to Ti-10Mo-xZr alloys. Phases transformation BCC → HCP occurred at 754 °C and 582 °C for 0Zr and 30Zr wt%, respectively, with a reduction ≈ of 170 °C. For 0Zr% alloy, the difference between transition temperature points at BCC_B2 and HCP_A3 lines ≈ is 970 °C, but for 30Zr%, this difference is approximately 1040 °C. This increase represents the temperature ranges where the β phase stabilizes; this range increased with Zr addition, claimed to be a β-stabilizing element. The intermetallic phase representing the Mo_2_Zr compound was obtained at low temperatures near 400 °C and identified later as C15_LAVES.

**Fig. 4:**
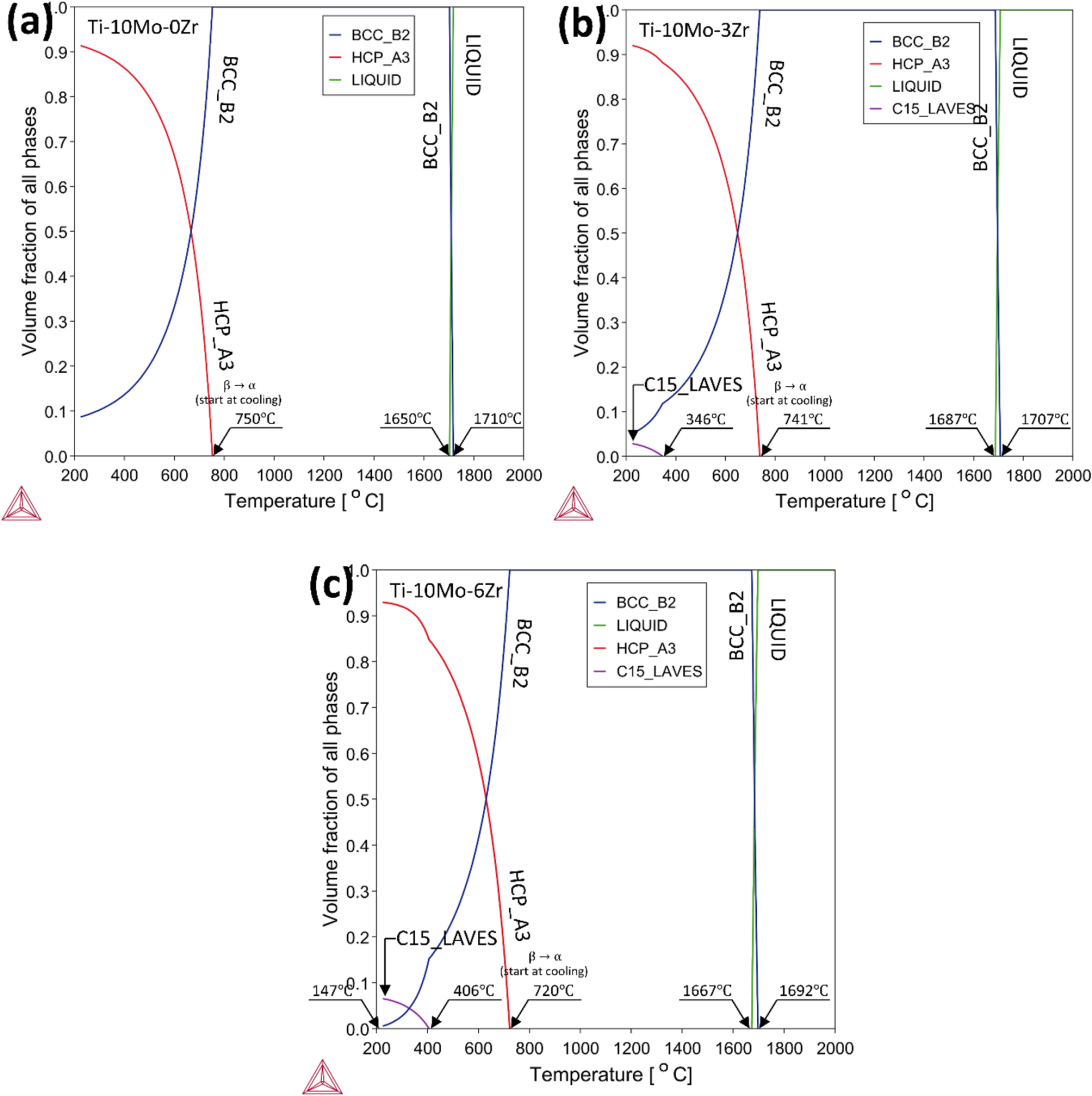
Molar contents illustrate different stabilized phases for TMZ0, TMZ3 and TMZ6 alloys versus temperature.

Figure 4 presents the volume fraction of the entire stabilized phases of the Ti-10Mo-xZr alloys as the Zr = x (0,3,6) wt% versus temperature. All alloys showed both β (BCC_B2) and α (HCP_A3) phases with approximate behaviors but different transformation temperatures and various volume fractions. β transus temperature according to β (BCC_A2) → α (HCP_A3) phase transformation starts at 750 °C for Ti-10Mo, and adding 3% Zr negligibly decreases the transition temperature down to 741 °C. Superior Zr addition with 6% dropped more obviously the β → α transformation temperature to 720 °C; this influence of Zr addition to β-type Ti-alloys agrees with the previous study of Kim et al., who reported Zr addition suppresses β → α transformation temperatures weakly^64^. The solidification temperature intervals for 0Zr, 3Zr, and 6Zr wt% are (1650–1710) °C, (1687–1707) °C, and (1667–1692) °C, respectively as Zr addition suppressed the Liquid → β transformation range compared with Zr%. The C15_LAVES phase appeared at 3Zr and 6Zr wt% until 346 °C and 406 °C, respectively. The equilibrium window of the C15_LAVES phase widened by Zr addition.

### Non-recrystallization temperatures modeling (TNR)

In this work, the study scope discards the as-cast condition because of the presence of segregation and large grain size, which are not suitable for the bio-implant applications.

**Fig. 5:**
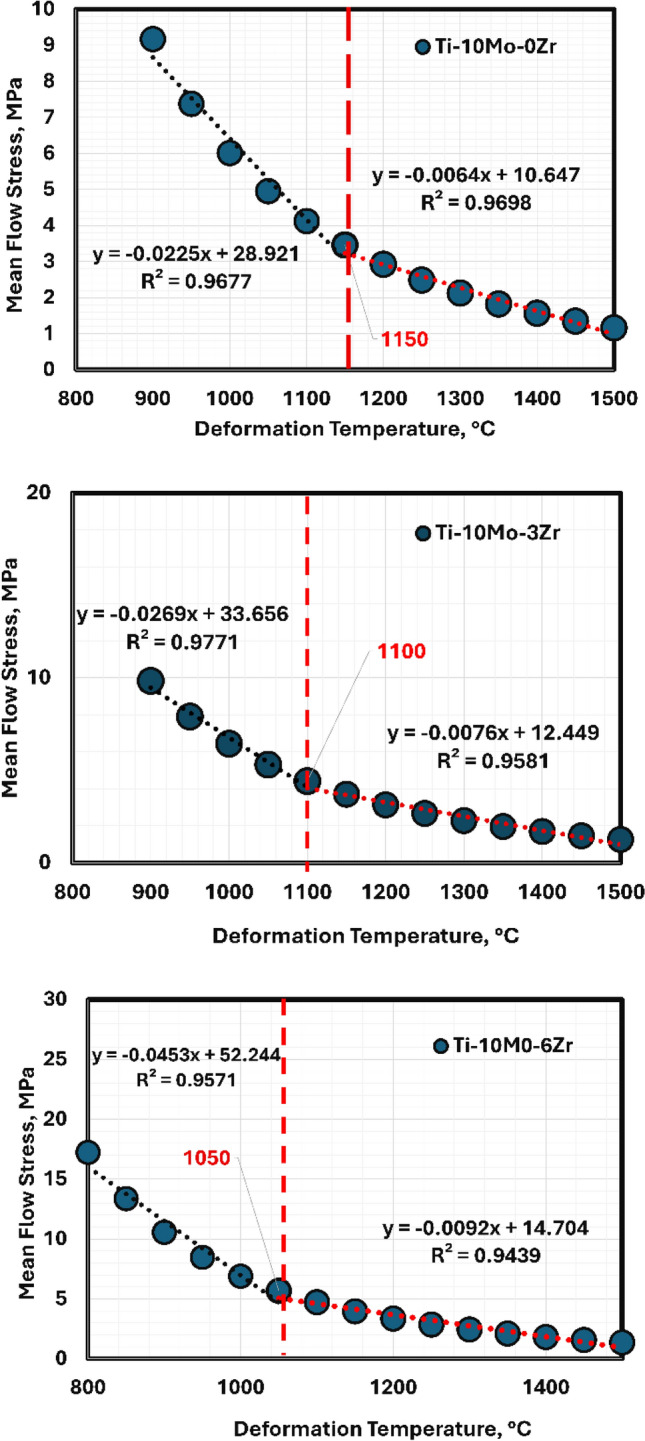
The relationship between mean flow stress and deformation temperature through hot forging using JMatPro software for TMZ0, TMZ3, and TMZ6 alloys.

**Fig. 6:**
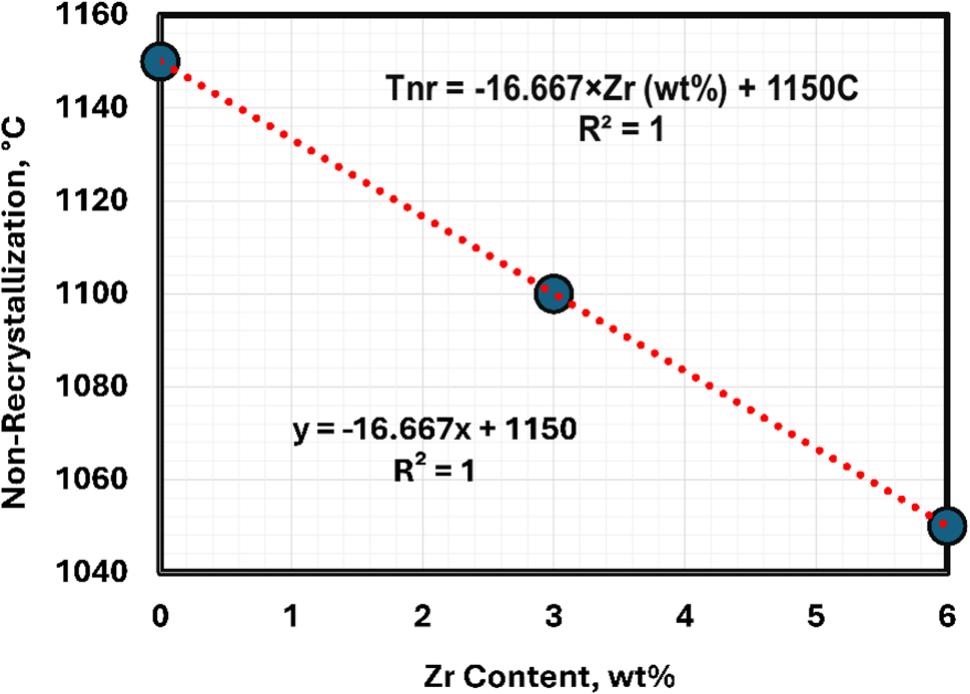
Non-recrystallization temperatures through hot forging using JMatPro software for TMZ0, TMZ3, and TMZ6 alloys.

The non-recrystallization temperatures (TNR) of the studied alloys were obtained through a modeling JMatPro software that simulated the hot forging process. The relation between the mean flaw stress and the deformation temperatures is presented in Fig. 5. The TNR temperatures are achieved through modeling with values 1150, 1100, and 1050 °C for hot forged TMZ0, TMZ3, and TMZ6 alloys, respectively, as shown in Fig. 6. TNR temperatures are essential for choosing the required thermomechanical treatment temperatures to achieve the required mechanical properties. The hot forging process applied in this study was below the TNR temperatures in order to control and avoid the grain growth and material recrystallization.

### Differential scanning calorimetry analysis (DSC)

**Fig. 7:**
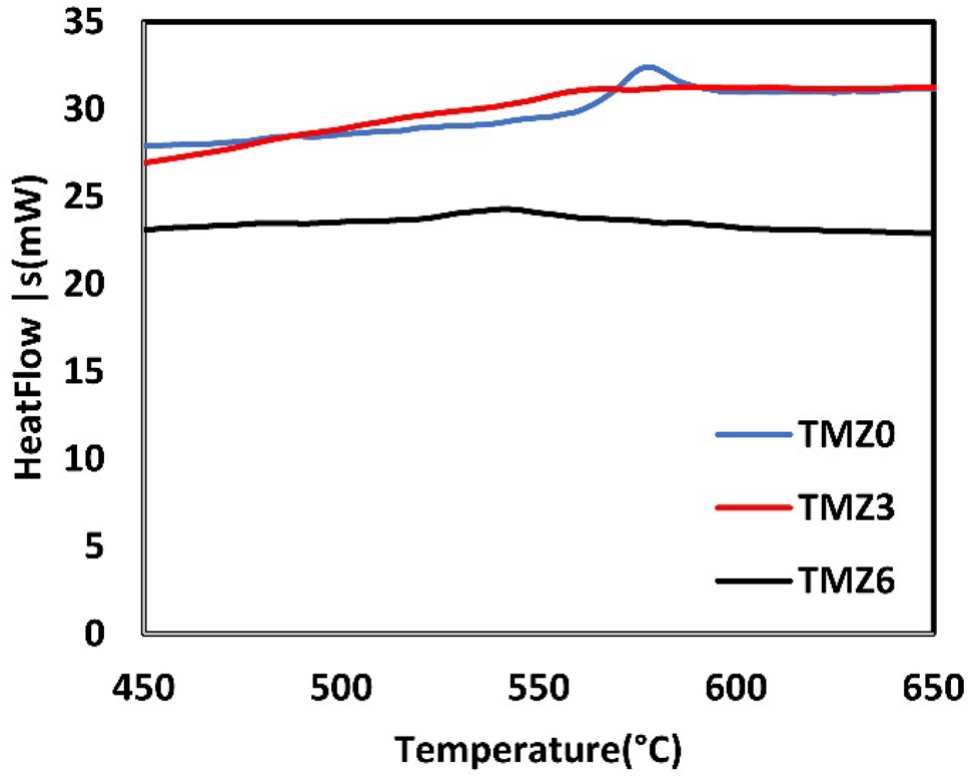
DSC curves of TMZ0, TMZ3, and TMZ6 alloys after the hot forging process using a heating rate of 10 °C/min.

The relationship between thermodynamic calculations done by the Thermo-Calc software approach and experimental results of phase temperature transitions will be discussed using the DSC test. DSC heating cycles for hot forged TMZ0, TMZ3, and TMZ6 alloys are plotted in Fig. 7. The exothermic peaks that appeared represent the regime phase transformation of α → β between 520 °C and 600 °C; the Zr addition decreased the height of the measured exothermic peaks. The α → β phase transition temperature decreased by Zr content; the transition ranges are as follows: 560–592 °C, 548–570 °C, and 523–560 °C for 0Zr, 3Zr, and 6Zr alloys, respectively. These results agree with Thermo-Calc calculations presented in Fig. 4, in which α → β phase transformation temperatures are 750 °C, 741 °C, and 720 °C for 0Zr, 3Zr, and 6Zr alloys, respectively. For both the Thermo-Calc approach and DSC results, Zr addition decreased the β transit temperature as Zr acted as a β-stabilizer element. There is a shift between Thermo-Calc calculations and experimental results because Thermo-Calc calculations assumed complete atomic diffusion, while β-transus measured temperatures depend on the atomic diffusivity^65^.

Considering non-equilibrium conditions at DSC measured, a calculation of ΔT expressing experimental correction is derived using the following formula^66^.

$$\Delta T = \, - 1.63*\left[ {Mo_{eq} } \right] + 20.14$$

**Fig. 8:**
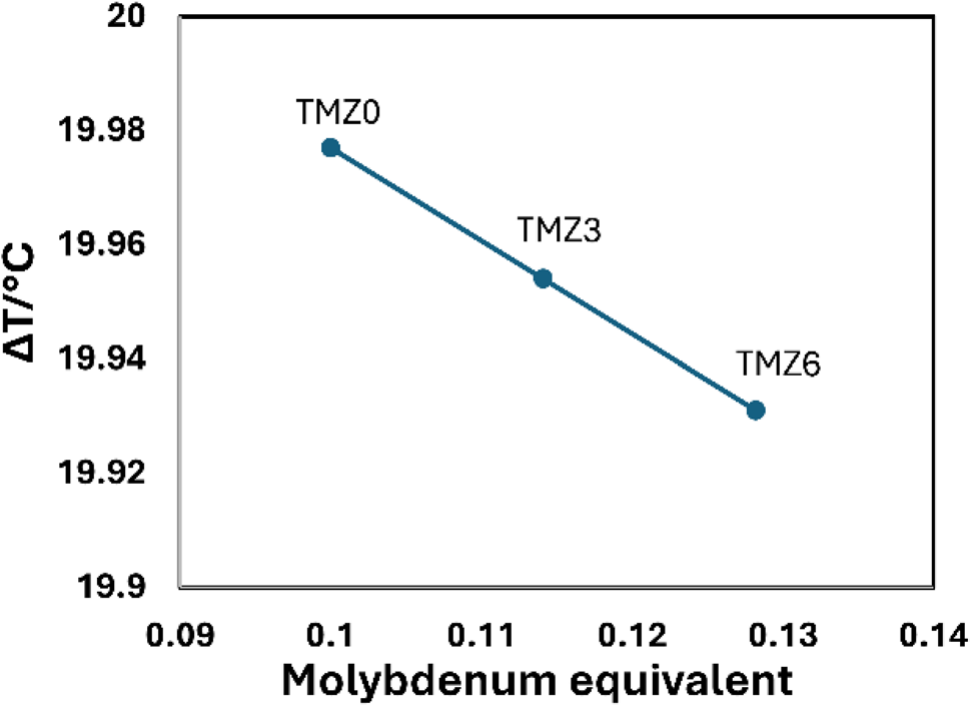
The relationship between ΔT and Moeq for TMZ0, TMZ3, and TMZ6 alloys after the hot forging process.

The relationship between ΔT and Moeq for TMZ0, TMZ3, and TMZ6 alloys is plotted in Fig. 8, where ΔT and Moeq show a linear relation. The Zr addition, which is a β stabilizer element, increased Moeq values and, subsequently, a significant decrease was observed in ΔT calculus. D.V.Gadeev et al.^66^ investigated the β-transus temperatures and found that a reduction in heating rate to zero eliminates heating rate errors and achieves equilibrium conditions.

### Microstructural analysis

**Fig. 9:**
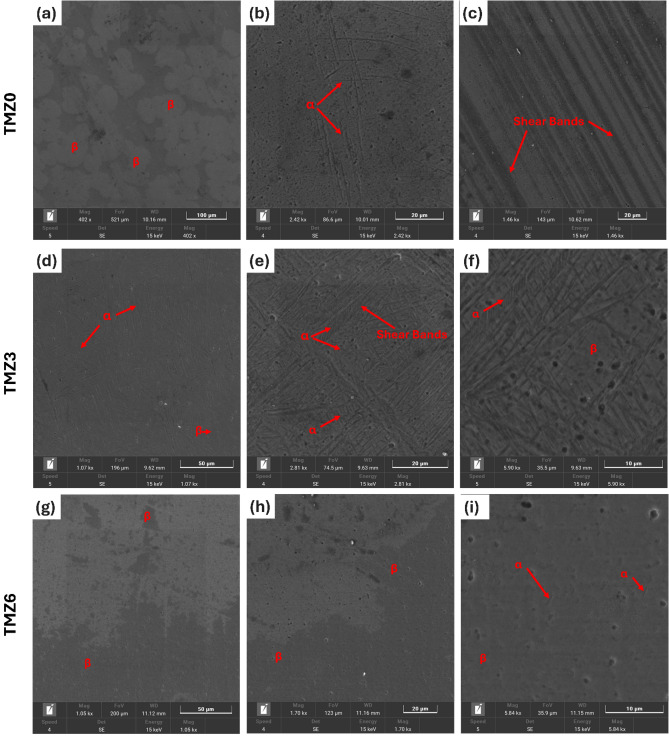
SEM images of (**a**) TMZ0, (**b**) TMZ3, and (**c**) TMZ6 alloys after the hot forging process.

The micrographs of all alloys using SEM are presented in Fig. 9. The microstructure showed both α laths and β grains, with the highest amount of α phase in the TMZ3 alloy. These results agree with the XRD results presented later in Fig. 12, in which the α phase percentage calculated through relative areas of phase peaks in the TMZ3 alloy was 52.5%. The thermo-mechanical treatment affects the formation of metastable transition phases, as W. Elmay et al. found that β phase transformation into an allotropic phase is induced because of the applied deformation stress^67^. Aline Nunes et. al^68^. made a comparison between different Mo addition percentages to the Ti element and found that starting with the Ti-10Mo alloy composition, the martensite phase α" is no longer favorable to precipitate; this finding agrees with the current study’s observed microstructure.

A previous study found that the formation of the β phase was delayed by the solution treatment effect for Ti-7.5Mo-xZr alloys compared with as-cast alloys^31^. D.R.N. Correa et al. studied the effect of alloying elements of zirconium and molybdenum on the microstructure and stabilized phase composition; they found that both elements are favorable to stabilize the β phase^41^. Thermo-Calc theoretical calculations, presented in Fig. 4, agree with those findings by D.R.N. Correa, as both considered zirconium as a β-stabilizer in Ti-alloys. Upon the previous discussion, a dual effect occurred by the thermo-mechanical treatment and zirconium element addition, with an opposed influence on β phase stability, explaining the current achieved results.

**Fig. 10:**
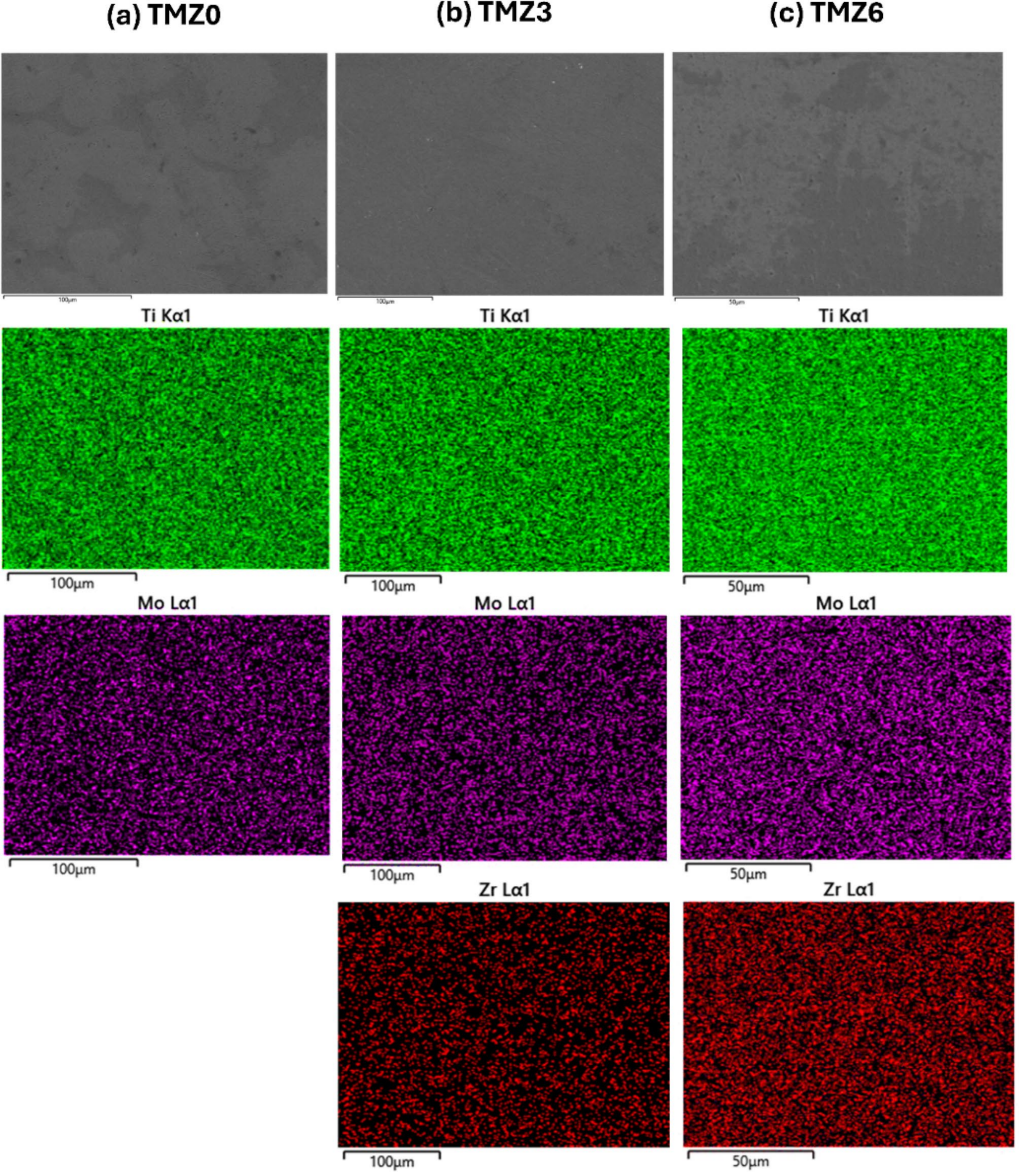
Elemental mapping of (**a**) TMZ0, (**b**) TMZ3, and (**c**) TMZ6 alloys after the hot forging process.

Figure 9 includes SEM images for hot-forged TMZ0, TMZ3, and TMZ6 alloys and typical micrograph images of hot-forged alloys containing both α + β phases. Thermo-mechanical treatment of the currently studied alloys produced initial coarse β grains; the deformation of previous alloys was not uniform, as deformation bands appeared near the grain boundaries in the TMZ0 alloy. These results agree with the previous study by S.L. SEMIATIN^69^, which studied α + β and β-type Ti-alloys applied to hot working having an initial microstructure including coarse β grains. The deformation investigated in the previous study was not uniform, as the deformation varied between near grain boundaries and grain centers, as achieved in the current work. The high deformation at β grain boundaries was produced by discontinuous dynamic recrystallization. Increasing the zirconium element amount in the TMZ6 suppressed α-phase precipitation. This agrees with the experimental results of XRD discussed later in Fig. 12, in which the α percentage intensively dropped for the TMZ6 alloy. Elemental mapping for hot forged TMZ0, TMZ3, and TMZ6 alloys is plotted in Fig. 10. The elements are distributed uniformly without any segregation.

### Phase identification results

**Fig. 11:**
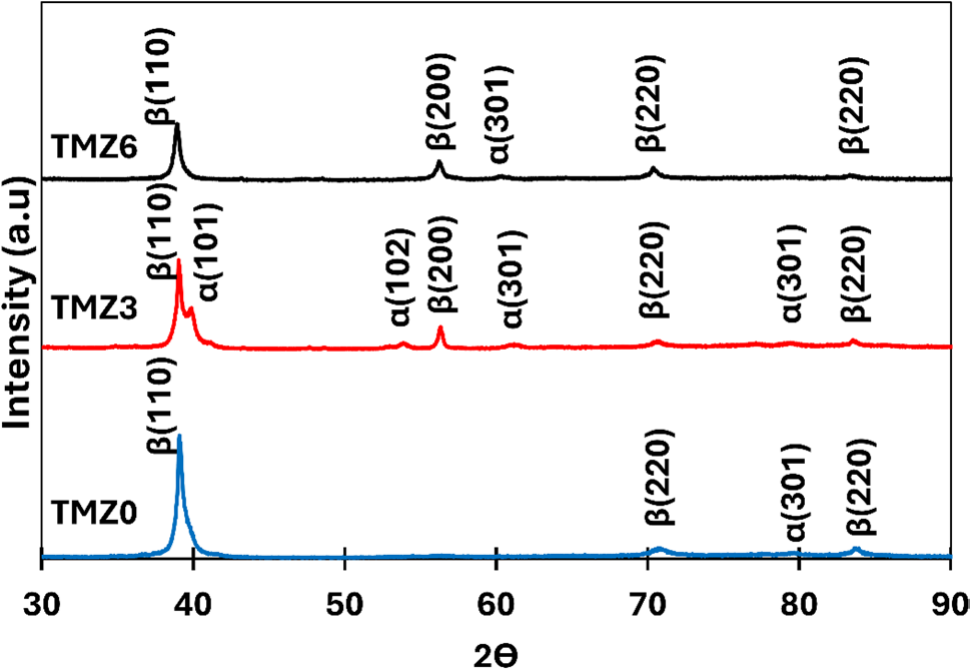
XRD curves of TMZ0, TMZ3, and TMZ6 alloys after the hot forging process.

**Fig. 12:**
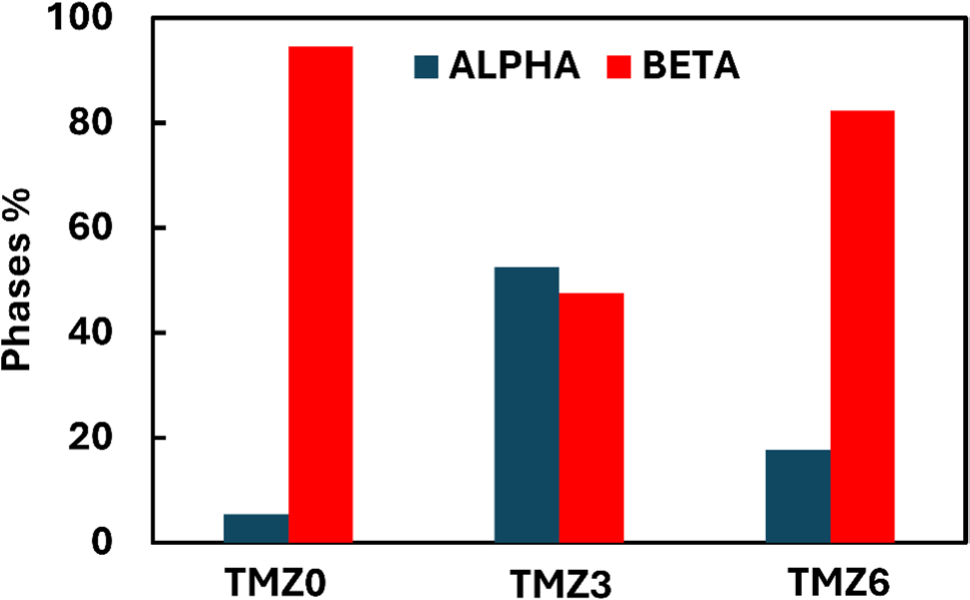
Phases precipitate percentages of TMZ0, TMZ3, and TMZ6 alloys after the hot forging process.

The phase precipitates of hot-forged Ti–10Mo–(x)Zr alloys were investigated using XRD analysis, as shown in Fig. 11. All investigated alloys were found to obtain both α + β phases with varied values. Also, the XRD did not detect the ω phase for any of the tested samples. From XRD patterns, the percentages of each α and β phase were calculated for TMZ0, TMZ3, and TMZ6 alloys and plotted in Fig. 12. The phase percentages were calculated using the area under the curve and the peak positions. The volume fraction of each phase was calculated by dividing the volume fraction of that phase (A_s_) by the sum of all phases’ volume fractions (ƩA_s_) using the following equation^22^:

$$Vfs = \frac{{As}}{{\sum As}}$$

The β phases decreased with the increase of the Zr element from 0 to 3%, as for the TMZ0 alloy; the predominant phase was β with an approximate percentage of 94.6%. But for TMZ3, β phase volume fraction percentage dropped down to 47.5%; this reduction in β phase amount is caused by the effect of thermo-mechanical treatment, as discussed in previous studies^31,67^.

These results agree with the SEM micrograph results discussed in Fig. 9, as TMZ0 and TMZ6 alloys have a small amount of α precipitates. For the TMZ3 alloy, the α phase is the predominant phase, despite TMZ0 and TMZ6 alloys, with a value of 52.5%. The formed phases mainly depend on the dual effect of thermo-mechanical processing and the effect of the Zr element in Ti-alloys as a weak β-stabilizing element. Ti and Mo elements have similar crystal structures and lattice parameters, resulting in the complete dissolution of each component into the other one; the mix of both elements produces a continuous solid solution at an elevated temperature above 900 ◦C upon the Ti-Mo binary phase stability diagram as previously studied^70^. The lattice parameter and comparison between the selective XRD peaks position of TMZ0, TMZ3, and TMZ6 alloys are listed in Table 3.

**Table 3:** Lattice parameters and comparison between selective XRD peaks position of TMZ0, TMZ3, and TMZ6 alloys after the hot forging process.

	Lattice parameter (cubic)				Lattice parameter (hexagonal)					
	a (Å)	β	2Ө	d (Å)	a (Å)	c (Å)	c/b	α	2Ө	d (Å)
TMZ0	3.2523	(110)	39.139	2.29	2.82	4.6	1.613	(101)	39.135	2.3
		(200)	56.549	1.62				(301)	79.703	1.2
		(220)	84.120	1.14						
TMZ3	2.9867	(110)	34.877	2.57	3.03	4.83	1.594	(101)	39.033	2.3
		(200)	62.105	1.49				(301)	79.703	1.2
		(220)	73.650	1.28				(102)	51.378	1.77
TMZ6	3.24	(110)	39.265	2.29	3.05	4.85	1.592	(101)	38.762	2.32
		(200)	56.739	1.62				(301)	67.812	1.38
		(220)	84.439	1.14				(102)	74.347	1.27

Investigating peak broadening of the XRD results, the lattice strain values that represent lattice distortion are generated; these Quantitative values are explained by the Williamson–Hall (W–H) equation^71^. Microstrains are generated from the full width at half maximum of XRD peaks (FWHM)^72^; G.K. Williamson and W.H. Hall created the Williamson-Hall method, which relies on size (Scherrer) broadening (βε) and strain broadening (β*D*) formulas, which vary with Bragg angle (θ) as follows;

$$\varepsilon = \beta \varepsilon /4 \, \tan \theta$$

$$D = K\lambda /\beta D\cos \theta$$

Using the former equations;

$$\beta tot = \beta \varepsilon + \beta D = 4\varepsilon \tan \theta + K\lambda /D\cos \theta$$

By multiplying Eq by cos(θ) :

$$\beta tot\cos \theta = 4\varepsilon \sin \theta + K\lambda /D$$

where D = grain size (determined from Williamson-Hall plot).

Comparing the last equation for a straight-line standard equation, y = mx + c, in which m = slope and c = intercept. The Williamson-Hall plot is derived from plotting βtot cosθ versus sinθ, and the strain is obtained from the slope of the linear line (4ε); the intercept *K*λ⁄*D* represents the size component^73,74^.

**Fig. 13:**
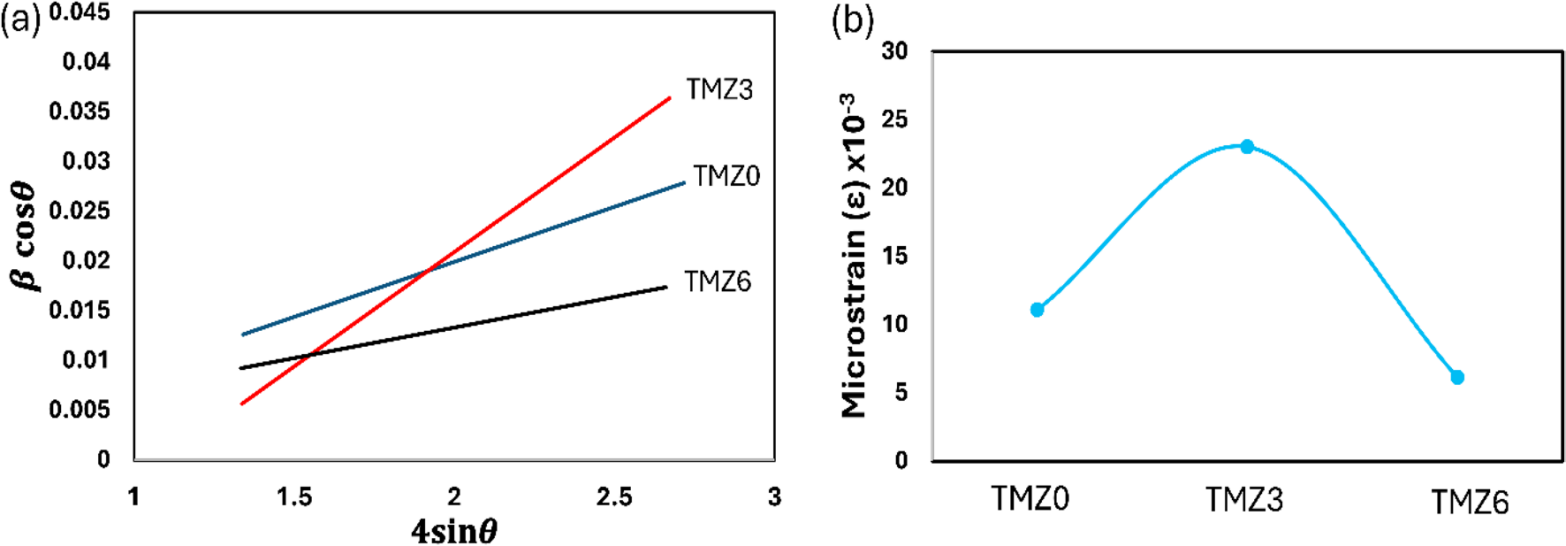
(**a**) Williamson–Hall plots, (**b**) Microstrain of TMZ0, TMZ3, and TMZ6 alloys after the hot forging process.

Figure 13a presents the Williamson–Hall plots derived from previous calculations; The linear fit results were used for calculating crystallite size from the y-intercept, and the microstrain resulted from the slope of the fit. The microstrain values plotted in Fig. 13b are 11.08*10^–3^, 23.01*10^–3^, and 6.13*10^–3^ for hot-forged TMZ0, TMZ3, and TMZ6 alloys, respectively. An earlier study found that the microstrain values achieved in Ti alloys are much higher for Zr-free ones^75^.

### Mechanical properties

The strength of Ti-alloys was reported to correlate with several factors, such as (1) Composition, (2) Applied processes, (3) Heat treatment conditions, and (4) strengthening mechanisms (precipitation hardening, solid solution strengthening, and grain refinement)^76^.

### Hardness

**Fig. 14:**
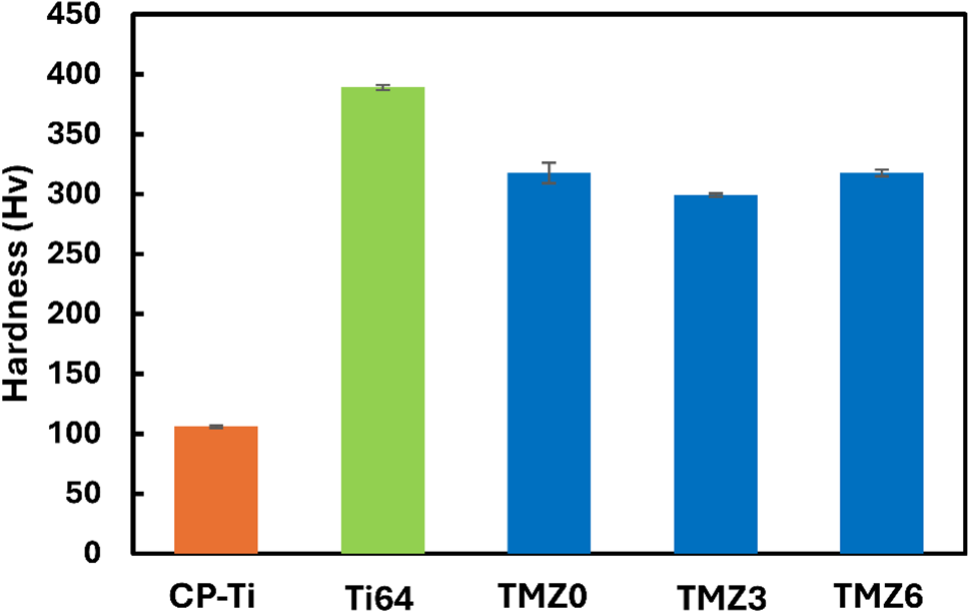
Hardness of TMZ0, TMZ3, and TMZ6 alloys after the hot forging compared with previous findings of CP-Ti and Ti64^79^.

A comparison between the hardness values of the currently studied alloys with commercial bio-implants, CP-Ti and Ti64 alloy from previous work^47^, is presented in Fig. 14. The average values of hardness are 317, 299.2, and 317 HV for 0Zr, 3Zr, and 6Zr alloys, respectively, with no significant difference. These findings agree with a previous study output in which Zr addition to Ti-14Mn-xZr with values 0Zr, 3Zr, and 6Zr wt% did not achieve a significant difference^77^. Both TMZ0 and TMZ6 alloys have almost the same average values of hardness, and both alloys achieved a high percentage of β-phase, where the predominant phase was β. Previous work showed that the hardness values depend on the phase stability, as it alters with the amount of α and β phases^34^. Caio Castanho et al.^78^ studied the effect of heat treatment on the Ti-15Zr-xMo alloys and derived the relation between the amount of β-phase and hardness, which exhibited a directly proportional relationship. The studied alloys achieved hardness values around triple that of CP-Ti, causing a better mechanical stability during wear exposure. In contrast, Ti64 alloy achieved a higher hardness, representing a better expected wear resistance.

Deformation patterns around the Vickers hardness notch indentation can give an approach to the entire deformation behavior, as it represents the elastoplastic behavior of metals^80^. These deformation bands could be in the form of either straight or wavy lines beside the notch indentation of the hardness test. The borderline between slipping and twinning in the $\overline{Bo }-\overline{Md }$ diagram can be determined by observation of previous deformed bands, in which a straight line represents twinning. However, wavy bands occur due to slipping, as described in many studies^81^. Pile-up, sink-in, and cracks are the expected morphologies detected around the micro-hardness indentation^82^.

**Fig. 15:**
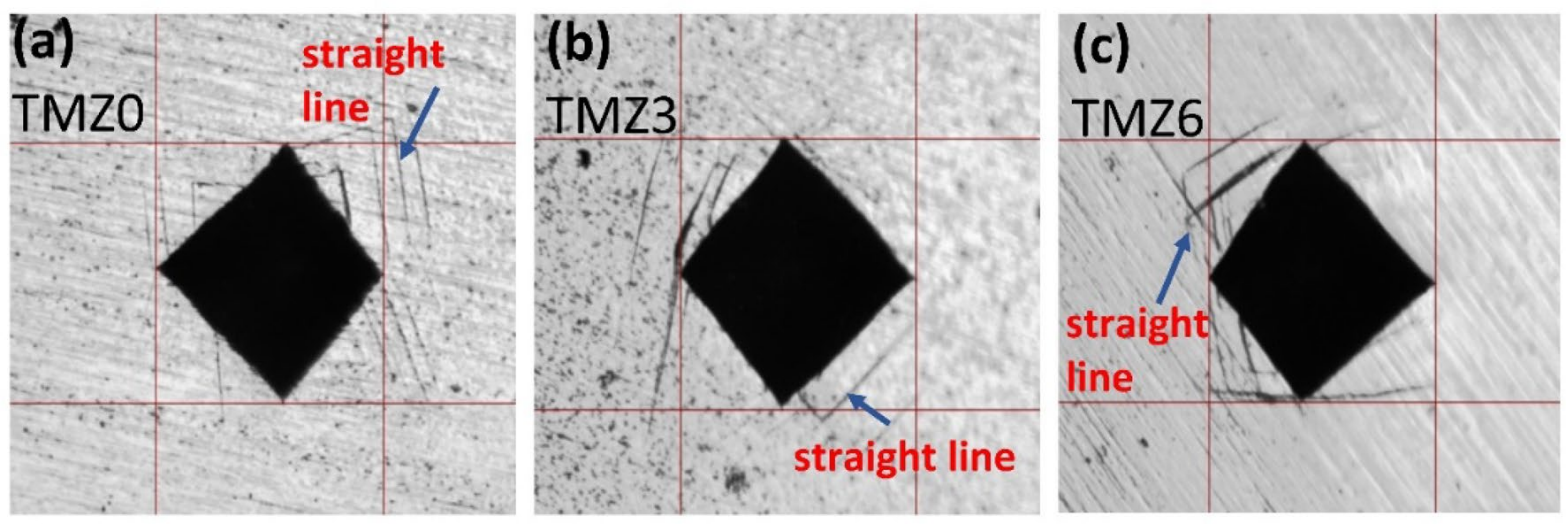
Hardness notch indentation of (**a**) TMZ0, (**b**) TMZ3, and (**c**) TMZ6 alloys after the hot forging process*.*

TMZ0, TMZ3, and TMZ6 alloys formed straight lines around the Vickers hardness indentation, as shown in Fig. 15. These straight lines give the approach of the entire deformation mechanism, as they represent twinning deformation. These results agree with the expected behavior estimated in the alloy design in $\overline{Bo }-\overline{Md }$ diagram and $\stackrel{-}{e/a}$- Δ $\overline{r }$ graphs in which all studied alloys are located in the twinning region, as discussed in the previous work^52^. The TMZ0 alloy showed the highest number of straight lines compared with the other alloys; this could be referred to as its position in the $\overline{Bo }-\overline{Md }$ diagram^52^, which is located at approximately the borderline of twinning. All the indentations showed that the pile-up morphology observed in Fig. 15 is caused by high ductility; this agrees with the hardness results, Fig. 14.

### Modulus of elasticity

The Young’s Modulus variation is crucial for biomedical implant applications; hence, Young’s Modulus is inversely proportional to material spring back. However, the high spring back of bio-implants minimizes the stress-shielding effect on patient bones after surgery; subsequently, a low Young’s Modulus is required to adapt human bones mechanically. The Young’s Modulus of human bone averages between (10–30 GPa), so Ti alloys are the best choice for surgeons due to their changeable Young’s Modulus covering implant inquiries. The hot forged exhibits the highest E-modulus as atomic reduced by thermomechanical stress, resulting in higher bond strength^83–85^.

**Fig. 16:**
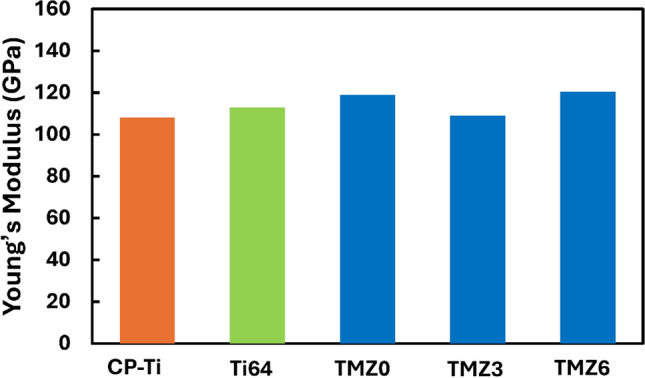
Young’s modulus (E) of TMZ0, TMZ3, and TMZ6 alloys after the hot forging process compared with previous study findings of CP-Ti and Ti64^90^.

The measured Poisson ratio values are as follows: 0.319, 0.338, and 0.336 MPa for 0Zr, 3Zr, and 6Zr alloys, respectively; the slight difference can be neglected. The E-Modulus slightly decreased by adding 3Zr to the investigated Ti-10Mo alloy, and then increased. The obtained E-Modulus measurements are 118.8, 108.9, and 120.4 GPa for 0Zr, 3Zr, and 6Zr alloys, respectively, as shown in Fig. 16 and Table 4.

**Table 4:** Mechanical properties of TMZ0, TMZ3, and TMZ6 alloys after the hot forging process measured using a digital ultrasonic flaw detector.

	Poisson ratio (ν)	*V*_*L*_ (mm/μS)	*V*_*S*_ (mm/μS)	G (GPa)	K (GPa)	E (GPa)
TMZ0	0.3192	5.89	3.034	45.038	109.6	118.83
TMZ3	0.3383	5.869	2.901	40.697	112.3	108.93
TMZ6	0.31654	5.955	3.085	45.74	109.42	120.43

E-Modulus depends on the phase percentages of α and β and strengthening mechanisms upon Zr addition. Several studies focused on the effect of phase stability on Young’s Modulus, β-type Ti alloys reported comparable lower Young’s moduli as Eβ < < Eα^18,86–88^. The bound energy of atoms is affected by increasing amounts of elements added to alloys; subsequently, superior atomic diffusion attenuation and dislocation movements occur, showing increases in the entire Young’s Modulus^31^. E. A. Trofimov et al.^89^ studied the separate α and β phases’ elastic modulus by using nanoindentation of commercial Ti64 alloy and achieved values of Eα = 92.8 ± 10.6 GPa and Eβ 75.8 ± 12.9 GPa; the grain size was inversely proportional to the elasticity modulus values.

Wen-Fu Ho^90^ studied Ti-Mo alloys and compared the results with the cast CP-Ti and Ti64 alloy. This study linked the stable phases with the E-modulus values, as CP-Ti includes only α-phase, while Ti64 alloy is composed of α + β phases. The TMZ3 alloy achieved almost the same E-modulus value as CP-Ti, as the TMZ3 alloy contained a high α%, as discussed previously in Fig. 12. Subsequently, the TMZ3 alloy will obtain the lowest amount of stress shielding effect, giving it an advantage in the mechanical compatibility with human bones. The studied alloys’ E-modulus value varied around that of the Ti64 alloy, with a slight difference representing alternatives to the common commercial alloys with better biocompatibility, as no toxic elements were included. The total range of Young’s modulus values for all alloys is between 108 and 120.4 GPa; this range is lower than that of conventional stainless steel and Co-Cr bio-implant alloys^91,92^.

### Compressive strength

All the studied alloys showed both α + β phases after thermo-mechanical treatments of TMZ0, TMZ3, and TMZ6 alloys, as discussed in the microstructural analysis sector of this work in Fig. 9. The analysis of XRD micrographs in Fig. 11 showed different phase percentages of α + β, in which β phase approximate percentages were 94.6, 47.5, and 82.3 for 0Zr, 3Zr, and 6Zr alloys, respectively. The variant of stabilized phases resulted from the dual effect of Zr addition and the influence of thermo-mechanical treatment. This variant of phases affects both yield strength and hardness, as well as the impact of Zr element addition in increasing strength.

**Fig. 17:**
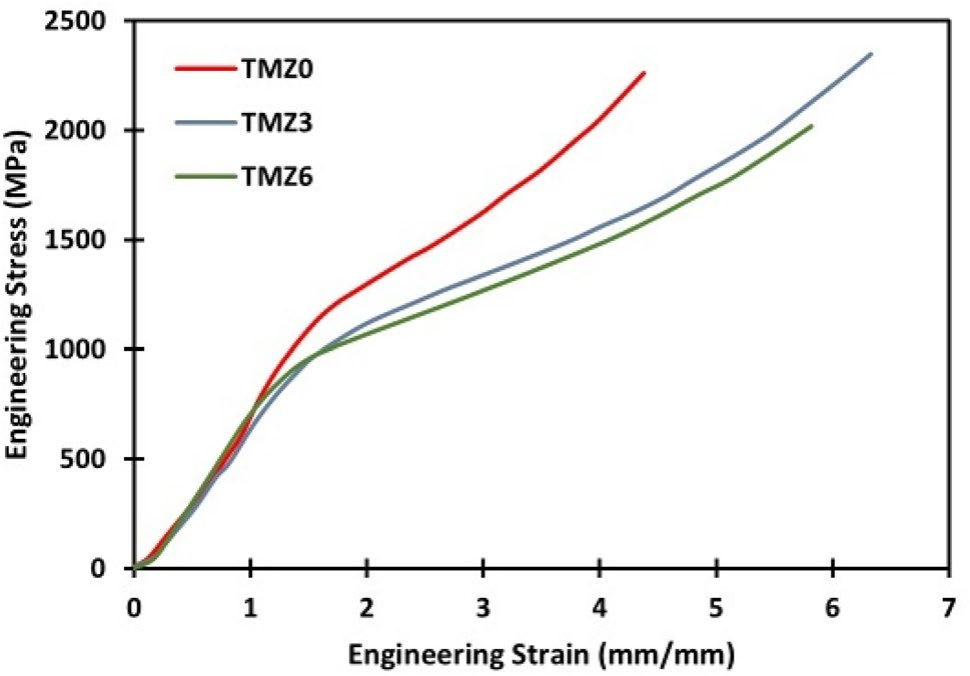
Engineering stress–strain curves of TMZ0, TMZ3, and TMZ6 alloys after the hot forging process obtained from compression tests at room temperature.

**Fig. 18:**
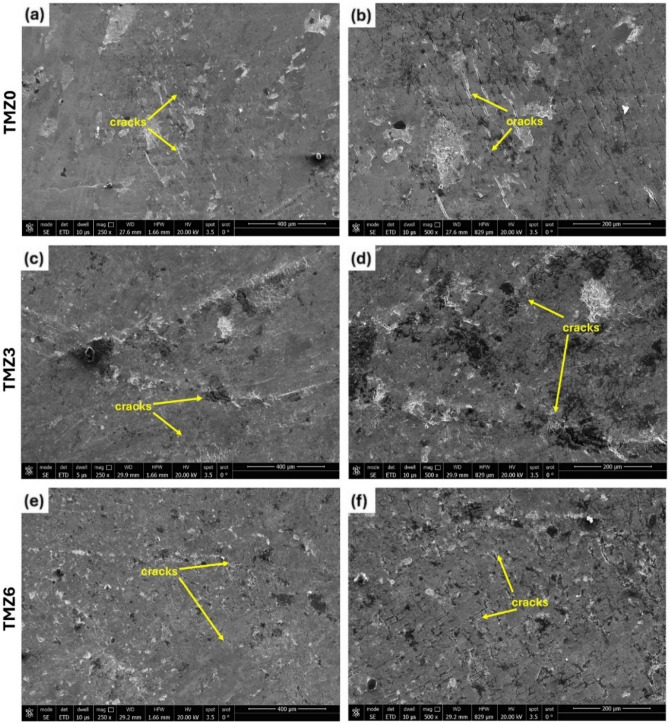
SEM micrographs of compression specimens of TMZ0, TMZ3, and TMZ6 alloys after the hot forging process.

Figure 17 presents the result for engineering stress–strain curves plotted during compression tests of the currently studied alloys at room temperature. All alloys showed similar behaviors in compressive; the yielding point ranges are as follows: 1116, 977, and 960 MPa for 0Zr, 3Zr, and 6Zr alloys, respectively, and achieved high malleability above 60%. Zr theorized that lattice distortion can be induced through the inhibition of dislocation movement, resulting in a strength increase^47^. From literature research, by comparing the current study results with the commercial Ti64 bio-implant under variant conditions, for annealed Ti64 (ΔL/Lₒ≈25.6%, σ_0.2_ = 978 MPa)^93^, cast with isostatically hot-pressed treatment (σ_0.2_ = 897 MPa)^94^. A previous study on Ti-12Mo alloy found that the addition of the Zr element improved the yield strength and enhanced the ultimate compression strength^75^, similar to the present results. Also, the compressive material properties are related to the influence of several factors, including grain size, phase amounts, porosity amounts, dislocations, and entire defects, and solid solution strengthening^75^. The surface of compressed specimens of hot forged TMZ0, TMZ3, and TMZ6 alloys after the compression tests is plotted in Fig. 18. Circumferential cracks appeared on all outer surfaces; the height of compressed specimens decreased to 60% without failure.

### Corrosion in simulated body fluid media

**Fig. 19:**
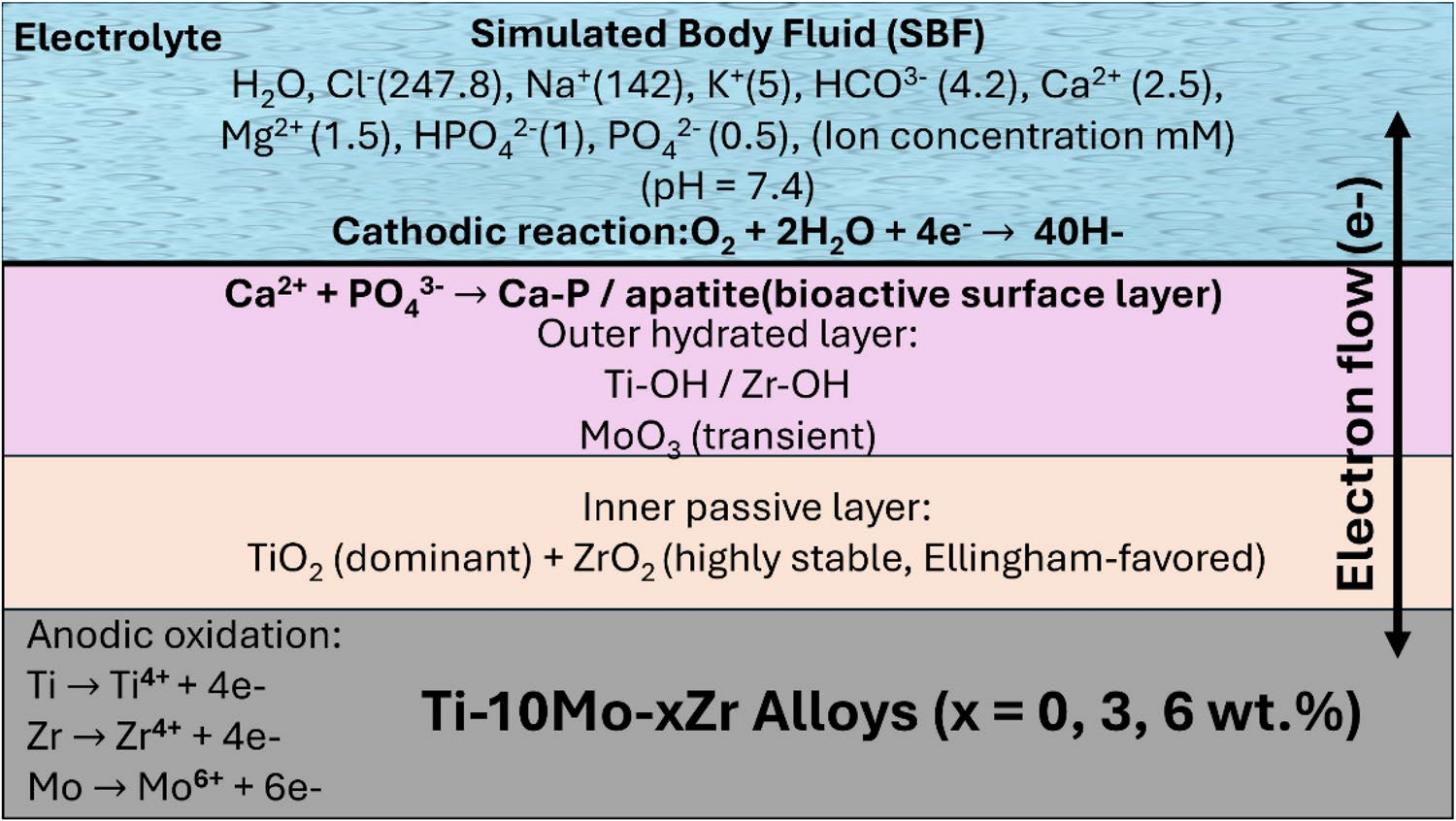
Suggested mechanism of electrochemical corrosion of TMZ alloys. Data adapted from Ref.^99–101^.

Figure 19 shows a suggested schematic illustration of the electrochemical corrosion and passivation mechanisms of TMZ alloys in simulated body fluid (SBF). When the TMZ alloy is exposed to SBF, anodic oxidation of the metallic elements occurs at the metal/electrolyte interface, with anodic reactions (oxidation): Ti → Ti4 + + 4e-, Zr → Zr4 + + 4e-, and Mo → Mo6 + + 6e-^95,96^. These metal cations rapidly react with oxygen or hydroxyl ions present in SBF to form protective oxides. In near neutral, the cathodic reaction is oxygen reduction (reduction): O2 + 4H + + 4e − → 2H2O (acidic) and O2 + 2H2O + 4e − → 4OH (neutral/alkaline). Titanium ions hydrolyze rapidly to form titanium dioxide (TiO2: primarily rutile or anatase), which forms a dense, adherent, and highly stable passive film that acts as the primary corrosion barrier, as per the following equations: Ti4 + + O2 − → TiO2 and/or Ti4 + + 2H2O → TiO2 + 4H +. Also, Zirconium contributes to passivity through the formation of zirconium dioxide (ZrO2), which is thermodynamically stable, electrically insulating, and enhances the compactness and stability of the passive layer, as per the following equations: $Zr4 + \, + \, O2 - \, \to \, ZrO2 \, and/or \, Zr4 + \, + \, 2H2O \, \to \, ZrO2 \, + \, 4H +$^95,97^. Moreover, Molybdenum oxidizes to form MoO3 molybdate species, where Mo-rich oxides improve corrosion resistance by increasing repassivation kinetics and suppressing localized breakdown in chloride-containing environments such as SBF, as per the following equations: $Mo6 + \, + \, 3H2O \, \to \, MoO3 + 6H +$^98^. The release of hydrogen ions leads to a catalytic process that helps to stabilize the formed oxide layers. After that, the alloy surface is covered by a multilayer passive structure consisting of an inner TiO₂, ZrO₂ barrier layer and an outer hydrated oxide region enriched in Ti–OH, Zr–OH, and transient Mo oxides. Zr seems to form a stable passive layer (ZrO₂) after the initial reduction to hinder further corrosion as compared to the other compositions. The high thermodynamic stability of TiO₂ and ZrO₂, as predicted by Ellingham diagrams, prevents the formation of unstable corrosion products^99^.

**Fig. 20:**
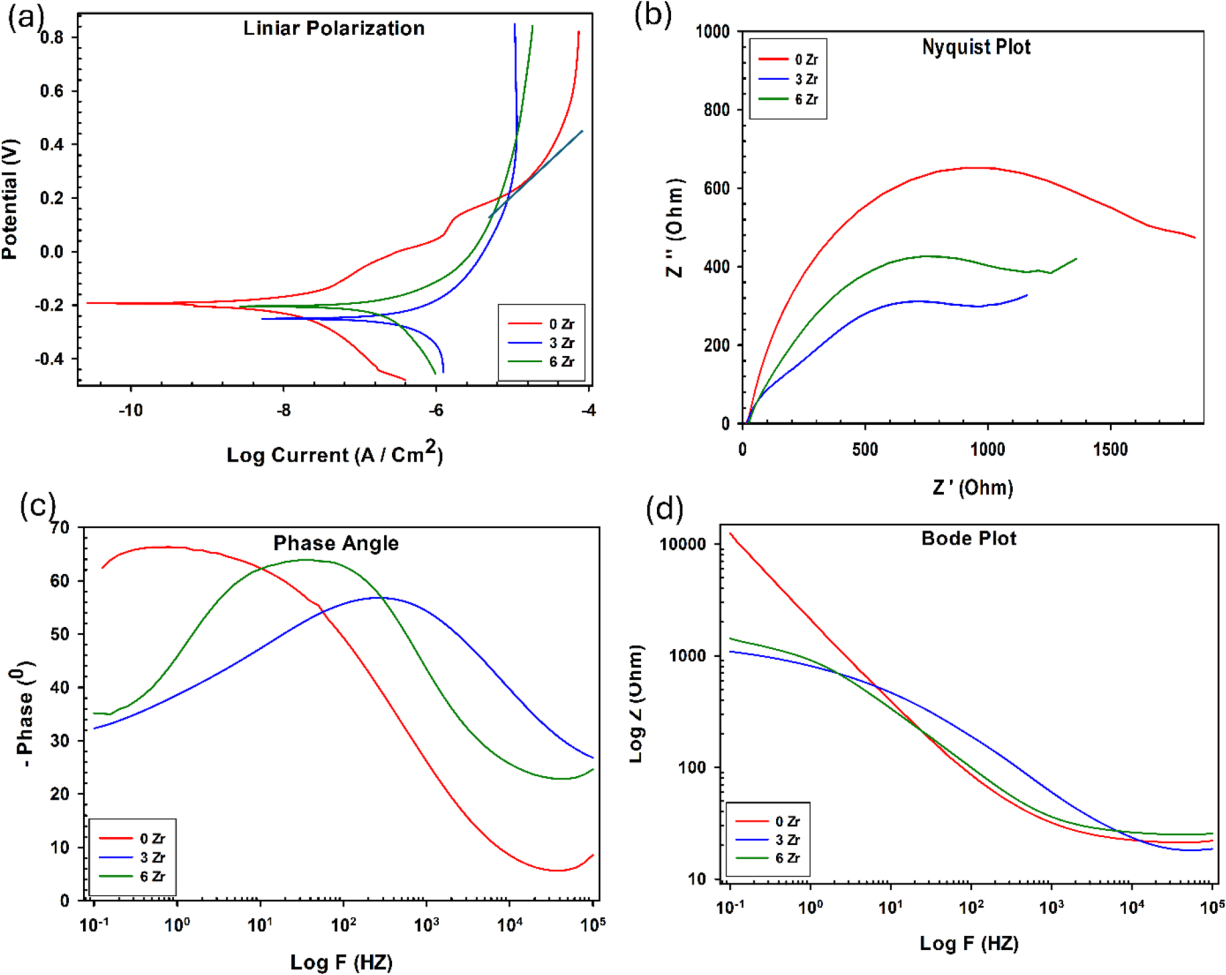
(**a**) Potentiodynamic polarization, (**b**) nyquist plot, (**c**) phase angle, (**d**) bode plot of TMZ0, TMZ3 and TMZ6 alloys after hot forging process tested in the SBF media*.*

The corrosion behavior for TMZ0, TMZ3, and TMZ6 alloys in simulated body fluid was studied at room temperature. The corrosion results achieved are listed in Table 5. Closed activation-controlled manner displayed in all examined alloys at the cathodic zone; the resulting linear polarization, in addition to electrochemical impedance spectroscopy (EIS) curves, is presented in Fig. 20. Linear polarization is plotted in Fig. 20a. The examined surfaces had no pitting, as the samples had passive behavior. Rapid potential increase observed for all studied alloys started at 280, −80, and −20 mV for TMZ0, TMZ3, and TMZ6 alloys, respectively. The TMZ0 alloy achieved the highest corrosion resistance value, with an annual corrosion rate of 0.21843 × 10^–3^ mm/year, and TMZ0 exhibits the lowest icorr with a value of 3.335 × 10^–10^ A/cm^2^. The dual effect of the thermomechanical treatment and Zr addition contrast influence on the amount of phases resulted in the non-linear behavior.

**Table 5:** Electrochemical parameters of TMZ0, TMZ3, and TMZ6 alloys after the hot forging process tested in SBF media.

Alloy	E_corr_ (mV/cm^2^)	i_corr_ (A/cm^2^) × 10^–10^	R_s_(Ω)	R_p_(Ω)	N	Q (μMho × S^N^)	CR(mm/ year) × 10^–3^
TMZ0	−3.105	3.335	114.99	68,428	0.78945	23.3	0.21843
TMZ3	−3.831	67.99	139.54	1178.2	0.79417	104	4.5714
TMZ6	−3.368	9.375	118.6	1187.2	0.61567	106	0.642

Electrochemical Impedance Spectroscopy (EIS) charts are presented in Fig. 20, The Nyquist plot supports the formation of a protective and passive layer as an incomplete semicircle arc shape observed for TMZ0, TMZ3 and TMZ6 alloys, as shown in Fig. 20a. The straight line of the TMZ0 alloy compared to the other alloys indicates more favorable corrosion resistance behavior. The phase shift and Bode plot versus the frequency range are presented in Fig. 20b,c. TMZ0 and TMZ6 alloys under examination displayed capacitive behavior for the passive along the frequency range from 0.1 to 1000 Hz. For TMZ0, TMZ3, and TMZ6 alloys, the phase angle value is near −67, −57, and −64◦, respectively. For TMZ0, at frequencies (log F) greater than 10^4^ Hz, the phase angle approaches zero, resulting in the impedance plateau and electrolyte resistance.

**Fig. 21:**
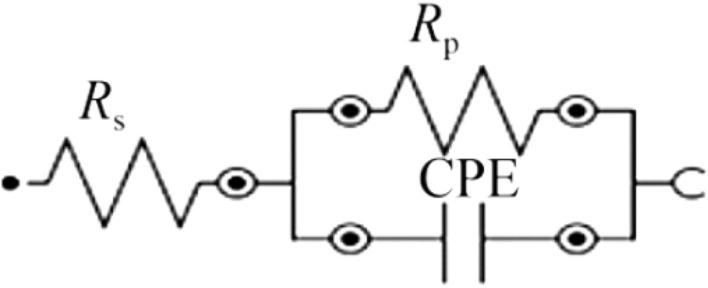
Randles circuit of TMZ0, TMZ3, and TMZ6 alloys used for corrosion testing in saline media.

The Randles circuit applied in the corrosion testing is shown in Fig. 21, which displays a better analysis of the physical behavior matching with the EIS spectra. The electrochemical parameters of electrolyte resistance (Rs), polarization resistance (Rp), constant phase element(Q), and ideal factors (n) are compared in Table 5. The Rs of the tested alloys in SBF fell within a narrow range of 114.99 to 139.54 Ω. The passive layer’s capacitance was reported with values less than optimum in the 0.615–0.794 n range as (n < 1). The Rp values are inversely proportional to icorr as well as corrosion rate (CR). TMZ0 alloy displayed the highest corrosion resistance with an Rp value equal to 68,428 Ω. In contrast, the TMZ3 alloy exhibits the lowest Rp value equal to 1178.2 Ω and the highest icorr reported from the potentiodynamic tests.

## Conclusion

This study investigated the dual effect of Zr element addition as well as the thermo-mechanical processing during hot forging of Ti-10Mo-xZr alloys on the material stabilized phases, mechanical properties, and corrosion behavior. The output conclusions are as follows;

- β-transus temperature declines upon Zr element addition to Ti-10Mo alloy, which was approved using Phase diagram calculations by Thermo-Calc software and DSC experimental results.
- Thermo-mechanical treatment, as well as Zr addition to Ti-10Mo, were the factors affecting the phases’ stabilization behavior.
- For both Ti-10Mo and Ti-10Mo-6Zr alloys, the predominant phase was the β phase with values of 94.6% and 82.3%, respectively. For the Ti-10Mo-3Zr alloy, the α phase accounted for a value of 52.5%.
- The studied alloys’ E-modulus value varied around that of the Ti64 alloy, with a slight difference representing alternatives to the common commercial alloys with better biocompatibility, as no toxic elements were included. The Ti-10Mo-3Zr alloy achieved almost the same E-modulus value as CP-Ti, with the lowest value of 108.93 GPa.
- The total range of Young’s modulus values for all alloys is between 108 and 120.4 GPa; this range is lower than that of conventional stainless steel and Co-Cr bio-implant alloys.
- Mechanical stability of the current studied alloys during wear exposure is much higher than that of CP-Ti due to the significant difference in micro-hardness results. In contrast, Ti64 alloy reported a higher micro-hardness, representing a better expected wear resistance.
- Alloys with a high β-phase fraction achieved better corrosion resistance, as the Ti-10Mo alloy has the lowest corrosion resistance, with an icorr value of 3.335 × 10^–10^ A/cm^2^. In contrast, the Ti-10Mo-3Zr alloy with the highest α-percentage exhibited the highest corrosion rate, as the icorr value equal 67.99 × 10^–10^ A/cm^2^.

## Data Availability

Data are available from the corresponding author, Alaa Keshtta, upon reasonable request.
